# *QuickStats*: Percentage* of Adults Aged ≥45 Years Who Reduced or Delayed Medication to Save Money^†^ in the Past 12 Months Among Those Who Were Prescribed Medication, by Diagnosed Diabetes Status and Age^§^ — National Health Interview Survey, 2015

**DOI:** 10.15585/mmwr.mm6625a5

**Published:** 2017-06-30

**Figure:**
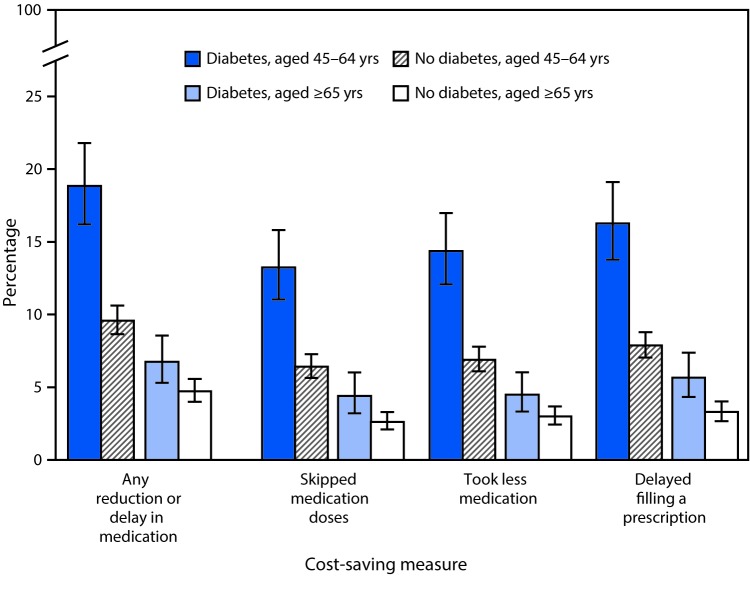
In 2015, among adults aged 45–64 years who were prescribed any medication, those with diabetes were more likely than those without diabetes to have reduced or delayed medication (18.8% compared with 9.6%) to save money in the past 12 months, with measures that included skipping medication doses (13.2% compared with 6.4%), taking less medication (14.4% compared with 6.9%), and delaying filling a prescription (16.3% compared with 7.9%). Among adults ≥65, those with diabetes were more likely than those without diabetes to reduce or delay medication (6.8% compared with 4.7%) and to have used each of the specific cost-saving measures. Regardless of diabetes status, among adults who were prescribed medication, those aged 45–64 years were more likely than those aged ≥65 years to reduce or delay taking medication to save money.

**Source:** National Health Interview Survey, 2015 data. https://www.cdc.gov/nchs/nhis.htm.

